# Decentralised health planning under National Rural Health Mission in Bihar, India

**DOI:** 10.1186/1753-6561-6-S1-P11

**Published:** 2012-01-16

**Authors:** Ajit Kumar Singh, Rashi Jayaswal

**Affiliations:** 1 National Health System Resource Center, New Delhi, India; 2 State Health Society, Department of health and family welfare, Bihar, India

## Introduction

The National Rural Health Mission (NRHM) proposed the decentralisation of health planning so that the state health plan represents the needs and priorities of respective blocks and districts in the state. In Bihar, the State Programme Implementation Plan (SPIP) for the year 2010-2011 has been framed on the basis of strategies and activities, which worked in the last four years. State has identified major bottlenecks and attempted to overcome them through alternative strategies.

In this paper we describe in detail the processes and outcomes realised in achieving decentralised planning in Bihar.

## Methods

The state constituted planning teams and designated nodal officers at block and district levels for preparation of SPIP under NRHM. District planning team was constituted by assistant chief medical officer (ACMO), district programme manager, district accounts manager, one district programme officer, one medical officer in-charge (MOIC), and one block health manager. Two Intensive (seven days) capacity building workshops were conducted for district planning team with support from national health system resource centre, public health resource centre, and state programme officers of state health society and government of Bihar.

Block planning team was constituted by the MOIC, block health manager, and block accounts manager. At the district level ACMO and at the block level MOIC and different district programme officers were designated as nodal officers for planning exercise in district and various blocks.

Resource package was communicated to all the districts and blocks based on the district and block fund allocation in previous year with an anticipated 25% increase from previous year's budget allocation. Furthermore, the financial guidelines prepared by respective state programme officers were disseminated to districts and blocks.

Districts thereafter conducted capacity building workshops for the blocks. Situational analysis and consultative workshops were conducted at block level to formulate the block health action plan, which was then sent to district. District health action plans were prepared by consolidating block plans and incorporating district level requirements/priorities, identified through district level situational analysis and consultations. Districts presented their plans to the respective SHSB officials/state programme officers at a state level workshop and subsequently finalised the plans considering the feedback received.

Information/data for planning process were gathered from primary and secondary sources. Focus group discussions, interactions, and meetings in different districts formed the primary source of information on views of health workers and private partners. Reports, registers as well as independent national surveys (e.g. national family health surveys, national sample surveys etc) formed secondary sources of information.

## Results

State health society, under the guidance of secretary of health has brought in a systemic change in the planning process incorporating the principle of decentralised planning advocated by NRHM. For the financial year 2010-2011, the district health action plans as per the NRHM guidelines have been prepared for 32 out of 38 districts. Planning processes for the rest of the districts would to be completed by the end of January 2010. The state health action plan for the year 2010-2011 reflects the outcomes of the district health action plans.

It is for the first time in Bihar that the planning exercise has been undertaken at block levels resulting into district health action plans proposing district specific initiatives (See Table 1).

**Table 1:** Specific initiatives proposed in district health action plans

Proposed specific initiatives	Name of the district
Free ambulance services for pregnant women	Gaya
Blood donation camp	Gaya
Monthly village health and nutrition days at Aganwadi centre	Kishanganj, Nalanda and Nawada
Maternal death audit	Arwal, Bhagalpur, Buxar, Gaya, Jehanabad, Samastipur, Siwan, Vaishali and West Champaran.
Health camps through mobile medical units in Mahadalit Tola (Vulnerable groups)	Banka, Begusarai, Bhagalpur, Kaimur and Kishanganj
Training on medical termination of pregnancies and safe abortion to nurses, auxiliary nurse midwives, and medical officers	Aaria, Arwal, Aurangabad, Darbhanga, East Champran, Gaya, Gopalganj, Jamui, Jehanabad, Katihar, Khagaria, Kishanganj, Madhubani, Munger, Muzaffarpur, Nalanda, Nawada, Purnea, Rohtas, Samastipur, Sheohar, Siwan, Vaishali and West Champaran

## Discussion

Health planning experience in Bihar shows that it is possible to implement decentralised planning processes and facilitate formulation of health plans at different levels reflecting needs and priorities of those levels. Once the government of India approves Bihar SPIP for the year 2010-2011, the state propose to undertake the following steps;

• Uploading of documents regarding release of payments by government of India, Bihar state health plan, district health plans, as well as district fund allocations and financial guidelines on the website of state health society.

• Flexibility to districts to re-allocate funds within the sub-heads of the major budget sections, with the ceiling of annual target in order to enable needs based spending at district level.

• District level workshop with representation from the state health society (for clarity of process and guidelines) to finalise annual and quarterly fund allocation to blocks.

• State level officials to undertake activity-planning exercise covering the process indicators for each activity and time lines for completion of the same.

• State level workshop for development partners to ensure their support in proper implementation at the district and block level and to ensure optimum fund utilisation at the district level.

